# Prognostic Inflammasome-Related Signature Construction in Kidney Renal Clear Cell Carcinoma Based on a Pan-Cancer Landscape

**DOI:** 10.1155/2020/3259795

**Published:** 2020-04-03

**Authors:** Tianyu Zheng, Xindong Wang, Peipei Yue, Tongtong Han, Yue Hu, Biyao Wang, Baohong Zhao, Xinwen Zhang, Xu Yan

**Affiliations:** ^1^The VIP Department, School and Hospital of Stomatology, Liaoning Provincial Key Laboratory of Oral Diseases, China Medical University, Shenyang 110002, China; ^2^Department of Orthopedics, The First Hospital of China Medical University, Shenyang 110001, China; ^3^Department of Biochemistry and Molecular Biology, China Medical University, Shenyang 110001, China; ^4^Center of Implant Dentistry, School and Hospital of Stomatology, Liaoning Provincial Key Laboratory of Oral Diseases, China Medical University, Shenyang 110002, China

## Abstract

**Objective:**

To investigate the expression patterns and prognostic characteristics of inflammasome-related genes (IRGs) across cancer types and develop a robust biomarker for the prognosis of KIRC.

**Methods:**

The differentially expressed IRGs and prognostic genes among 10 cancers were analyzed based on The Cancer Genome Atlas (TCGA) dataset. Subsequently, an IRGs risk signature was developed in KIRC. Its prognostic accuracy was evaluated by receiver operating characteristic (ROC) analysis. The independent predictive capacity was identified by stratification survival and multivariate Cox analyses. The gene ontology (GO) analysis and principal component analysis (PCA) were performed to explore biological functions of the IRGs signature in KIRC.

**Results:**

The expression patterns and prognostic association of IRGs varied from different cancers, while KIRC showed the most abundant survival-related dysregulated IRGs. The IRG signature for KIRC was able to independently predict survival, and the signature genes were mainly involved inimmune-related processes.

**Conclusions:**

The pan-cancer analysis provided a comprehensive landscape of IRGs across cancer types and identified a strong association between IRGs and the prognosis of KIRC. Further IRGs signature represented a reliable prognostic predictor for KIRC and verified the prognostic value of inflammasomes in KIRC, contributing to our understanding of therapies targeting inflammasomes for human cancers.

## 1. Introduction

Inflammasomes are a kind of intracellular innate immune multiprotein complexes, the concept of which was introduced by Martinon in 2002 [1]. Inflammasomes consist of three components: sensor protein, apoptosis-associated speck-like protein containing a caspase recruitment domain (ASC)/PYCARD, and pro-caspase-1. Inflammasomes can be activated by recognizing pathogen-associated and damage-associated molecular patterns (PAMPs and DAMPs) via their sensor protein, inducing the activation of pro-caspase-1. Activated caspase-1 (CASP1) will promote the release of inflammatory cytokines interleukin (IL)-1*β* and IL-18 which subsequently participate in immune and inflammatory response [2]. Studies have shown that inflammasomes play essential roles in regulating the physiological and pathological processes and correlate with various human diseases such as type 2 diabetes [3], immune-related diseases [4], and tumor [5].

In tumor, inflammasomes prove to be double-edged. On the one hand, inflammasomes are involved in regulating antitumor immunity. Inflammasomes downstream effectors IL-18 and IL-1*β* can inhibit the killing against cancer cells by certain immune cells, which is detrimental to the control of tumor growth and metastasis [6, 7]. On the other hand, inflammasomes are critical in the regulation of multiple cell death modes such as apoptosis and pyroptosis. NOD-like receptor (NLR) containing a pyrin domain 3 (NLRP3) inﬂammasome and absent in melanoma 2 (AIM2) inﬂammasome can induce apoptosis by recruiting and activating caspase-8 via ASC [8]. In addition, inflammasome-mediated activated CASP1 can cleave gasdermin D (GSDMD) and expose N-terminus of pore-forming activity, leading to cell membrane nanopores and cell swelling, and finally to cell pyroptosis [9, 10]. Pyroptosis is a kind of programmed cell death marked by inflammatory cytokines release [11]. These inflammasome-mediated cell death pathways are undoubtedly beneficial to tumor inhibition.

Recent studies have demonstrated that the functions of inflammasomes in tumor, to a certain extent, are determined by the different types of cells and tissues [12–14]. However, there is still no systematic molecular profile of inflammasome-related genes (IRGs) across diverse human cancers until now. The accessibility of high-throughput expression datasets offers the opportunity to investigate the roles of inflammasomes in various cancers. In this study, we identified dysregulated IRGs and prognostic IRGs among 10 cancer types using transcriptome data from The Cancer Genome Atlas (TCGA) [15]. Kidney renal clear cell carcinoma (KIRC) was observed to have most significant IRGs dysregulation and association with tumor prognosis, but few studies have focused on the relationship between inflammasomes and KIRC.

KIRC, the most frequent type of renal cell carcinoma (RCC) [16], have high risk of metastasis and mortality [17]. Currently, the primary treatment for localized RCC remains surgery. However, occurrences of recurrence or distant metastasis in postoperative patients with KIRC account for approximately 30% [18]. Therefore, reliable prognostic models are urgently required to predict the risk of progression for patients with KIRC. From the perspective of pan-cancer analysis, the relationship between inflammasomes and KIRC might be quite close. Thus, an IRGs signature was further constructed to predict patient survival and detect the prognostic value of inflammasomes in KIRC. Broadly speaking, the pan-cancer analysis will help us better understand the molecular mechanism of inflammasomes in the progression of human cancers. Moreover, our robust prognostic indicator confirms the vital role of inflammasomes in KIRC and provides novel therapeutic strategies for KIRC.

## 2. Materials and Methods

### 2.1. Selection of IRGs

A total of 40 genes were included in the inflammasome-related gene set: 20 of them were retrieved from the gene set (REACTOME_INFLAMMASOMES, M1072) in the Molecular Signatures Database v7.0 [19, 20], while the added genes were described as the components of inflammasome complexes or being involved in the inflammasome-related pathways according to the published literature.

### 2.2. Samples of Databases

The RNA sequencing (RNA-Seq) cohorts and clinical information involved in the pan-cancer analysis were obtained from TCGA. To ensure the stability of differential analysis and survival analysis, we only selected 10 types of cancer containing more than 20 normal samples and 20 dead samples. The cancer types included colon adenocarcinoma (COAD), liver hepatocellular carcinoma (LIHC), breast invasive carcinoma (BRCA), head and neck squamous cell carcinoma (HNSC), lung squamous cell carcinoma (LUSC), lung adenocarcinoma (LUAD), kidney renal papillary cell carcinoma (KIRP), KIRC, stomach adenocarcinoma (STAD), and uterine corpus endometrial carcinoma (UCEC). For the TCGA cohorts, we downloaded counts as well as FPKM values of the mRNA expression data. The counts data were used for gene differential expression analysis, whereas FPKM data for prognostic genes identification.

For establishing the risk model in KIRC, 526 patients with complete survival information in TCGA_KIRC dataset were used as a discovery set. The FPKM values of the KIRC RNA-Seq were log-transformed by log_2_(FPKM+1) before being applied to the model. To test the prognostic reliability of the model, these 526 KIRC samples were randomized to internal validation set-1 (*n* = 132) or internal validation set-2 (*n* = 394). The TCGA database also included 72 normal samples, of which 71 had matched KIRC samples. Furthermore, three datasets (GSE40435, GSE53757, and GSE73731) were selected from the Gene Expression Omnibus (GEO) database as external validation cohorts for their larger sample sizes. In detail, GSE40435 contained 101 pairs of KIRC and the corresponding normal samples [21]; GSE53757 had 72 KIRC and 72 unpaired normal samples [22], whereas GSE73731 included 265 tumor samples only [23]. The expression matrixes of the GEO datasets were obtained and normalized by the limma package of R [24]. Above KIRC datasets and corresponding clinical features of patients are shown in Table 1.

### 2.3. Bioinformatic Analysis

The R package “edgeR” was performed to identify the differentially expressed IRGs between tumor and normal samples [25], with a filter condition of adjusted *p* value < 0.05 and absolute log fold-change (FC) > 1. Also, “edgeR” package was used to analyze differential expression between the high- and low-risk groups. The heatmap and principal component analysis (PCA) were carried out with R packages. The Search Tool for the Retrieval of Interacting Genes database (version 11.0) was used for accessing protein-protein interaction (PPI) [26] and Cytoscape software (version 3.7.2) for visualization [27]. To investigate the functional roles of the IRGs signature in KIRC, gene ontology (GO) analysis was conducted on g:Profiler database [28].

### 2.4. Statistical Analysis

Univariate Cox regression model was applied to obtain prognostic characteristics of IRGs. For constructing the IRGs signature in KIRC, least absolute shrinkage and selection operator (LASSO) Cox regression analysis was performed to select the most optimal prognostic genes by R package “glmnet” [29]. The packages “survival” and “survminer” of R were used for conducting Kaplan–Meier survival analysis and log-rank test to evaluate the survival difference. The “survivalROC” package was used to perform a time-dependent receiver operating characteristic (ROC) analysis, and the area under the curve (AUC) value was calculated to measure the prognostic accuracy of the risk signature. Additionally, univariate and multivariate Cox regression analyses were conducted to determine whether the risk signature was an independent prognostic factor for KIRC. The expression levels of unpaired samples and risk score distribution were evaluated by independent samples *t*-test, while the expression levels of paired samples were analyzed by paired *t*-test. All the statistical analyses were performed on GraphPad Prism 7 software (GraphPad Software Inc., La Jolla, CA) and R software (R.3.6.0). A two-tailed *p* value less than 0.05 was considered to be statistically significant.

## 3. Results

### 3.1. The Pan-Cancer Expression Patterns and Prognostic Characteristics of IRGs

**Figure 1:**
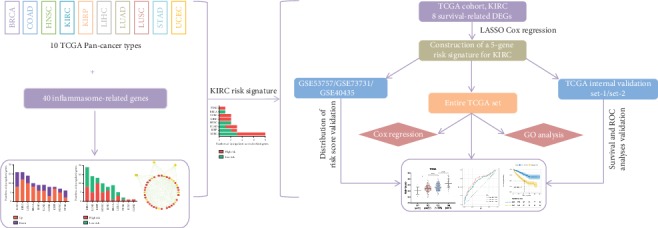
Flow chart of the analysis procedure: data acquisition, signature construction, and validation in KIRC.

**Figure 2:**
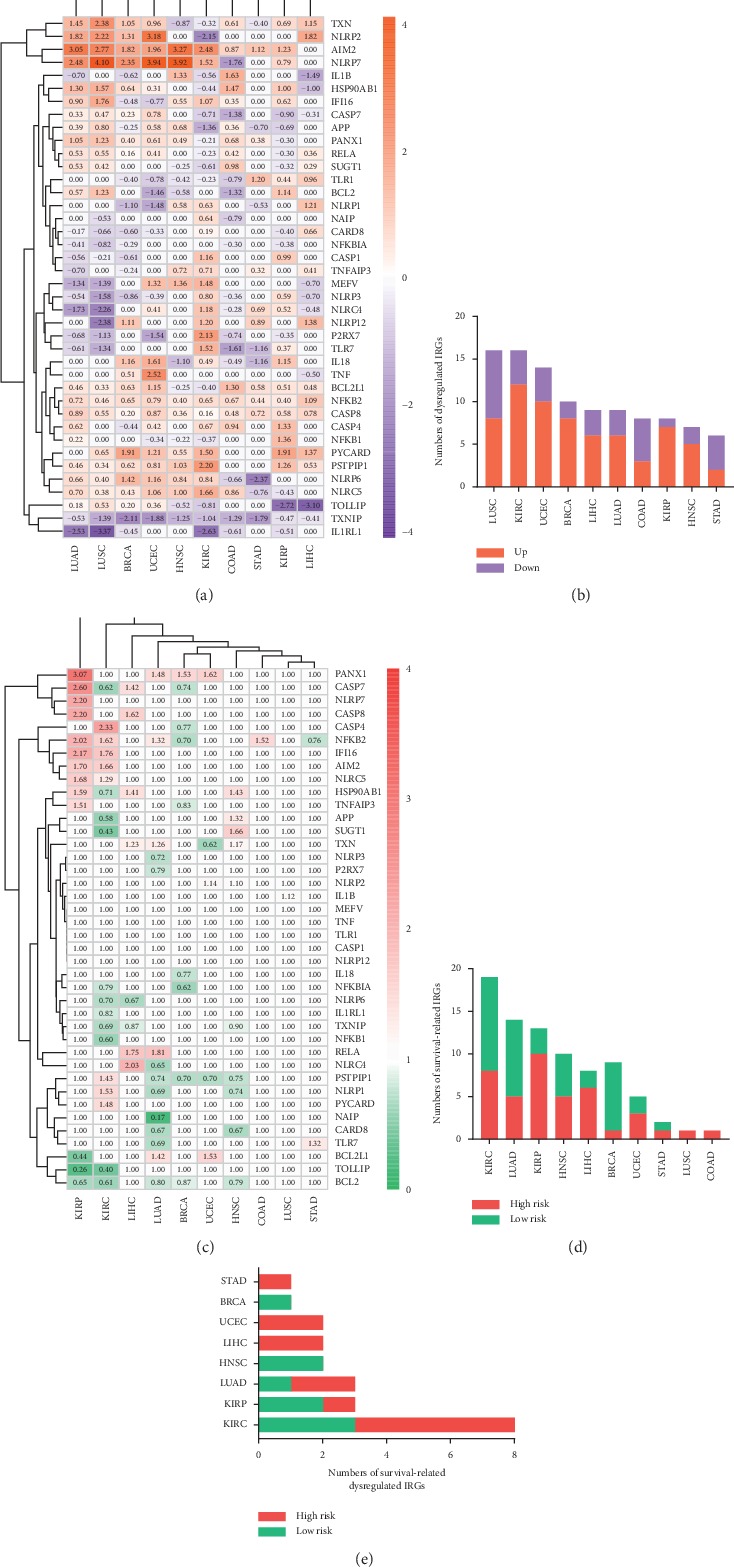
(a) The expression patterns of IRGs across cancers on transcriptome level. Numerical value reflects log FC. (b) The numbers of dysregulated IRGs among 10 cancers. (c) The association between IRGs and prognosis of cancers. Numerical value indicates hazard ratio (HR). (d) The numbers of prognostic IRGs among 10 cancers. (e) The numbers of dysregulated IRGs associated with survival among 8 cancers.

The flow chart of this study is shown in Figure 1. To explore the expression patterns and prognostic association of IRGs across human cancers, we identified differentially expressed IRGs between cancer and normal samples as well as survival-related IRGs among 10 cancers. Generally, 33 (82.5%) of the IRGs were dysregulated in one or more cancer types (Figure 2(a)), while 35 (87.5%) were significantly associated with overall survival (OS) of patients (Figure 2(c)). We observed AIM2 overexpression in 8 cancers and its association with poor prognosis in two kidney carcinomas (KIRC and KIRP). Thioredoxin-interacting protein (TXNIP) expression was consistently suppressed in 7 cancers and positively correlated with patient OS in 2 cancers (KIRC and HNSC) of the 7. The PPI network of all of the IRGs is displayed in Supplementary Figure 1. Furthermore, the expression patterns and prognostic characteristics of IRGs varied from different cancer types, and the numbers of dysregulated and survival-related IRGs for each type were calculated (Figures 2(b) and 2(d)). Abnormally expressed IRGs were most in LUSC and KIRC (*n* = 16, resp.) and least in STAD (*n* = 6). The unsupervised clustering showed that expression patterns of the homologous tissues LUAD and LUSC were relatively close (Figure 2(a)). Besides, survival-related IRGs were most in KIRC (*n* = 19) and least in LUSC as well as COAD (*n* = 1, resp.).

It is worth mentioning that prognostic and dysregulated IRGs were most abundant in KIRC, but rarely reported. Similarly, KIRC showed the largest number (*n* = 8) of dysregulated IRGs related to OS (Figure 2(e)). Furthermore, among the eight genes, the risky genes (PSTPIP1, IFI16, NLRC5, AIM2, and PYCARD) were consistently upregulated, whereas the protective genes (IL1RL1, TXNIP, and APP) were consistently downregulated in KIRC in comparison to normal tissues (Table 2). Due to most survival-related dysregulated IRGs and less studies of inflammasomes in KIRC, the prognostic value of inflammasomes in KIRC was worth investigating. Thus, we further developed an IRGs signature in KIRC to predict patient survival.

### 3.2. Construction of an IRGs Signature for Predicting OS of KIRC Patients

Based on the pan-cancer analysis, eight dysregulated genes associated with OS of KIRC patients were obtained from TCGA_KIRC dataset (Table 2). Then, a total of five prognostic genes were selected by LASSO Cox regression (Supplementary Figures [Supplementary-material supplementary-material-1] and [Supplementary-material supplementary-material-1]); interferon gamma-inducible protein 16 (IFI16) and AIM2 were identified as risky factors (HR > 1), whereas IL-1 receptor-like 1 (IL1RL1), TXNIP, and amyloid precursor protein (APP) were protective factors (HR < 1).

**Figure 3:**
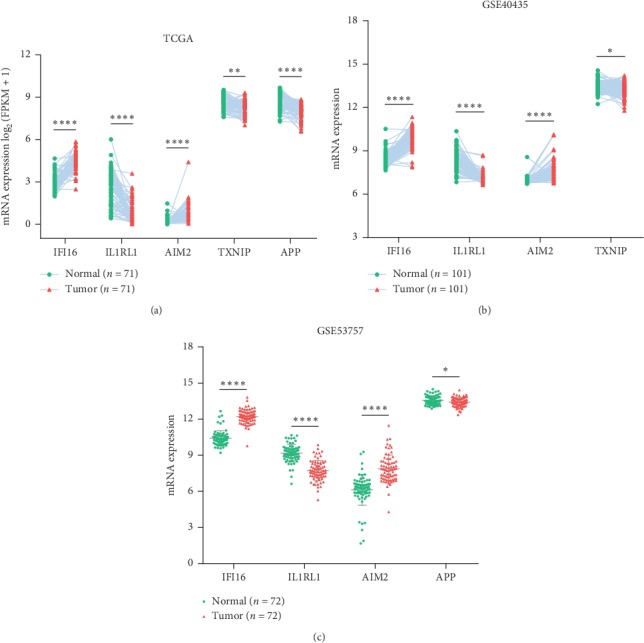
The mRNA levels of signature genes in paired KIRC samples (a-b) and unpaired KIRC samples (c). ^*∗*^*p* < 0.05, ^*∗∗*^ *p* < 0.01, ^*∗∗∗*^*p* < 0.001, and ^*∗∗∗∗*^*p* < 0.0001.

The five-gene differential expression in KIRC tissues was tested using paired KIRC samples from TCGA and GSE40435, as well as unpaired KIRC samples from GSE53757. As shown in Figure 3, RNA-Seq data from all these validated cohorts confirmed a significant dysregulation of IFI16, IL1RL1, and AIM2 in KIRC tissues. In addition, downregulation of TXNIP and APP was assured by two cohorts, respectively. Overall, compared with normal kidney tissues, the five genes were significantly dysregulated in KIRC. Subsequently, a risk signature was established based on the five IRGs' LASSO Cox regression coefficients (Table 2) and expression levels:

(1) $$\begin{array}{c} \text{Risk score}=\left(0.5409\times \text{IFI}16\text{ expression}\right)+\left(0.1449\times \text{AIM}2\text{ expression}\right)+\left(-0.0698\times \text{IL}1\text{RL}1\text{ expression }\right)+\left(-0.3261\times \text{TXNIP expression}\right)+\left(-0.5283\times \text{APP expression}\right). \end{array}$$

**Figure 4:**
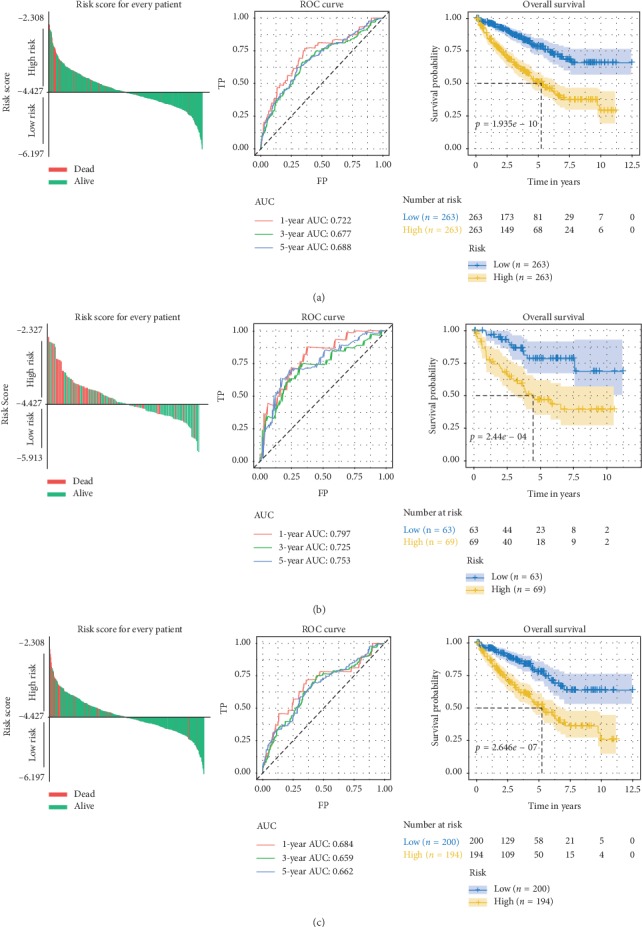
(a–c) Risk score for each patient, ROC curves (1-year, 3-year, and 5-year), and Kaplan–Meier survival curves in the entire TCGA set (a), internal validation set-1 (b), and internal validation set-2 (c).

The risk score for each patient was calculated by our IRGs signature. In the discovery set, patients were divided into high-risk and low-risk groups according to the median risk score of −4.427 as a cutoff value. The risk score, survival status, and the five-gene expression of each patient are displayed in Supplementary Figures [Supplementary-material supplementary-material-1]-[Supplementary-material supplementary-material-1]. As expected, the high-risk group had higher expression levels of the risky genes and lower expression levels of the protective genes (Supplementary [Supplementary-material supplementary-material-1]). Further, Kaplan–Meier analysis and ROC analysis were conducted in the discovery set (entire TCGA set), and high-risk group had significantly shorter OS time in comparison to the low-risk group (Figure 4(a)). ROC curves showed that the 1-year, 3-year, and 5-year predictive accuracy of the risk model were 0.722, 0.677, and 0.688, respectively (Figure 4(a)).

### 3.3. The IRGs Signature Associated with Poor Clinicopathologic Characteristics of KIRC Patients

**Figure 5:**
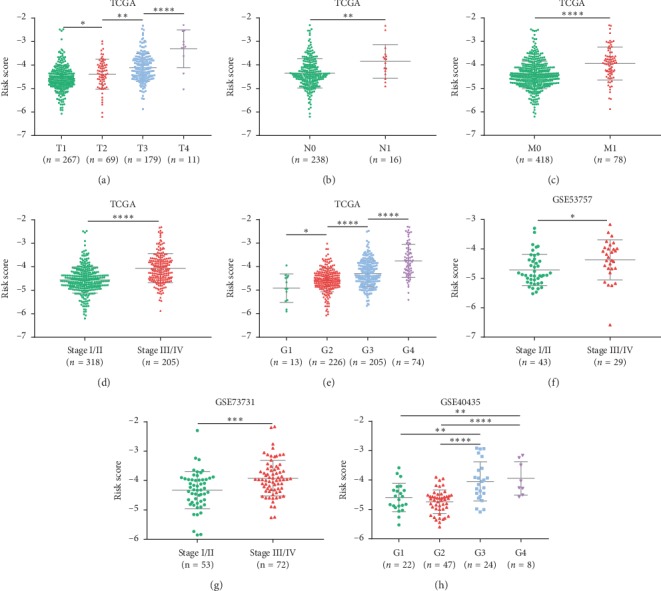
(a–e) Risk score distribution in the entire TCGA set by different T stages (a), N stages (b), M stages (c), TNM stages (d), and grades (e). Risk score distribution in the external validation sets by different stages (f-g) and grades (h).

Since the IRGs signature was negatively correlated to patient OS, we investigated its correlation with multiple clinicopathologic factors of KIRC patients based on the TCGA dataset. We compared distribution of the risk score among different tumor (T) stages, node (N) stages, metastasis (M) stages, TNM stages, and histologic grades. It was noteworthy that higher risk indicated more advanced grades of all these clinicopathologic parameters (Figure 5(a)–5(e)), suggesting the relationship between the IRGs signature and the progression of KIRC.

### 3.4. Validation of the IRGs Signature

To verify the reliability of our IRGs signature, two internal validation sets and three external validation sets (GSE40435, GSE53757, and GSE73731) were used to test above results. The same hazards model was applied to all the validation cohorts to obtain the risk score for each patient. In the internal validation set-1/set-2, the same cutoff value for grouping was utilized, and the prognostic association and predictive accuracy of the IRGs signature were assured (Figures 4(b)-4(c)). Besides, the findings that risk score related to stage and grade of KIRC were confirmed by the external validation cohorts (Figures 5(f)–5(h)).

### 3.5. Prognostic value of the IRGs Signature

**Figure 6:**
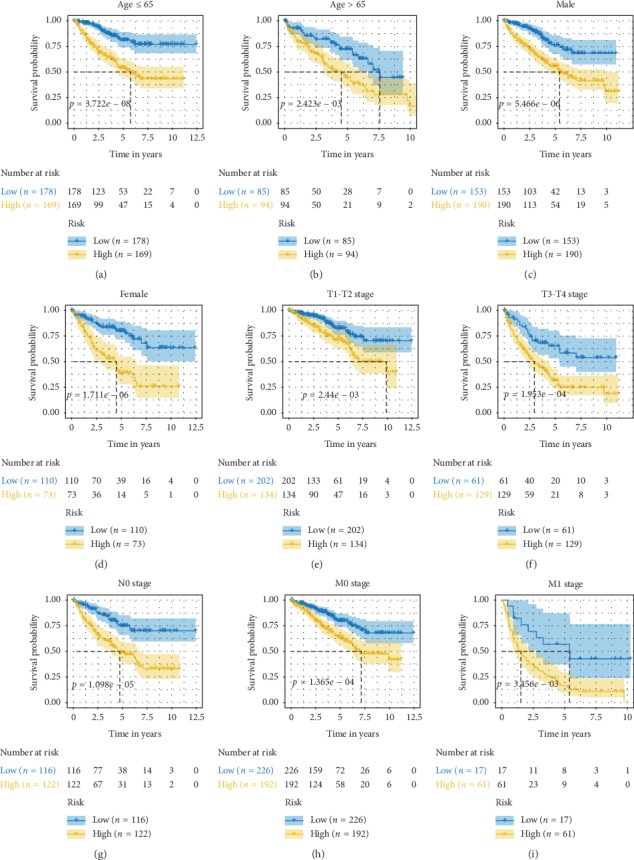
(a–i) Survival analyses of the IRGs signature in the TCGA cohorts stratified by age (a-b), gender (c-d), T stage (e-f), N stage (g), and M stage (h–i).

To detect the prognostic performance of the IRGs signature in stratified cohorts, patients were classified based on age, gender, tumor stage, lymph node status, and distant metastasis status. Due to the small sample size of patients at N1 stage (*n* = 16), we carried out stratification analysis on patients at N0 stage. In all cohorts, the high-risk groups were observed to have worse survival than the low-risk ones (Figure 6). Thus, the IRGs signature was able to distinguish patients with poor survival outcomes without considering traditional clinical factors. Additionally, univariate and multivariate Cox regression analyses were conducted in the entire TCGA set to explore whether the IRGs signature could independently predict OS for KIRC patients. As shown in Table 3, the IRGs signature remained significantly correlated with OS even adjusted by age, T staging, N staging, M staging, and grade, suggesting that the IRGs signature represent an independent prognostic predictor for KIRC.

### 3.6. Different Immune Response Patterns between High- and Low-Risk Groups

**Figure 7:**
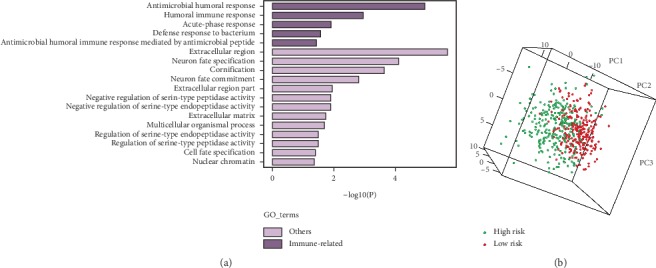
(a) GO analysis based on the upregulated genes (log FC > 2) in the high-risk group. (b) PCA analysis of the low- and high-risk groups based on immune-related genes in the entire TCGA cohort.

To unearth the biological characteristics of the IRGs signature in KIRC, we analyzed the differentially expressed genes between high-risk group and low-risk group using the entire TCGA dataset. Then, the significantly upregulated genes in the high-risk group (log FC > 2, adjusted *p* value < 0.05) were involved in the GO analysis. It was shown that immune-related pathways were enriched in the high-risk group (Figure 7(a)). We further conducted PCA of immune-related genes in the two groups, with the gene sets acquired from “Immune Process” and “Immune Response” GO terms. As a result, the high- and low-risk groups generally presented distinct directions of immune-related gene distribution, indicating different immune states of the two groups (Figure 7(b)). Above results indicated the five IRGs mainly involved in immune-related processes.

## 4. Discussion

Accumulating evidence has revealed that inflammasomes are involved in the pathological processes of different tumors [14, 30, 31], but the specific molecular mechanisms remained incompletely elucidated. In this study, we identified different expression patterns and prognostic characteristics of IRGs among 10 cancers. Remarkably, dysregulation and prognostic correlation of IRGs were most significant in KIRC. Meanwhile, KIRC had the most abundant dysregulated genes associated with patient survival. All of these suggested that inflammasomes might contribute to the progression of KIRC. Considering the potential essential roles of inflammasomes in KIRC and lack of relevant studies, we further focused the analysis on KIRC and established a risk signature for KIRC in order to guide the diagnosis and treatment of KIRC.

KIRC is an aggressive tumor that requires effective predictive biomarkers, but the prognostic models are currently limited. Based on the study value of inflammasomes in KIRC, we produced an IRGs signature to predict the prognosis of patients with KIRC. The signature gene dysregulation in KIRC was confirmed in both paired and unpaired tissues from TCGA and external validation cohorts, indicating the reliability of our differential analyses. Moreover, the fact that the five genes were consistently dysregulated in KIRC indicated the stability of our signature.

Survival analysis suggested that the IRGs signature was closely related to poor prognosis in KIRC. The ROC analysis showed that our signature had an accurate prognostic performance. Additionally, compared with several existing signatures for the TCGA discovery cohorts, our IRGs signature was demonstrated to have a superior predictive accuracy for 5-year survival (AUC = 0.688 vs. 0.637 [32]/0.649 [33]/0.660 [34]). We also performed Kaplan–Meier analysis in stratified cohorts and found its ability to identify patients with worse survival regardless of other clinical variables. Moreover, the five-gene signature was an independent prognostic indicator for KIRC according to the results of univariate and multivariate Cox analyses. Collectively, the IRGs signature can act as a reliable prognostic predictor for KIRC. The prognostic value of our IRGs signature verified the crucial roles inflammasomes played in KIRC, indicating IRGs as potential prognostic biomarkers for KIRC. Moreover, the five-gene signature was positively correlated with advanced stages of the clinicopathologic parameters, suggesting that the signature genes might impact the proliferation, metastasis, and differentiation of KIRC; nevertheless, previous studies have concentrated more on their functions in other cancers than KIRC; hence, further studies are needed for KIRC.

Among the five genes, both AIM2 and IFI16, as the PYHIN family members and innate immune DNA sensors [35, 36], were observed to be risky factors for KIRC. Interestingly, studies have previously reported the tumor-suppressive activity of AIM2 [37, 38] and IFI16 [39, 40]. However, increasing studies have also suggested their tumor-promoting property, corresponding to our findings. For instance, AIM2 improved proliferation of non-small-cell lung cancer (NSCLC) cells via inflammasome-dependent pathway [41]. Knockdown of either AIM2 or IFI16 in oral squamous cell carcinoma cells reduced cell growth [42]. As for the protective factors (IL1RL1, TXNIP, and APP), IL1RL1 was similarly identified to function as a tumor suppressor in mammary tumor [43]. TXNIP was commonly silenced in cancer cells due to genetic or epigenetic events [44]. In addition, a recent study revealed that downregulation of TXNIP could predict worse survival in KIRC, which is in good accordance with our results [45]. Regarding APP, current studies have demonstrated its overexpression and characteristic of oncogenes in some malignancies such as breast cancer [46], pancreatic cancer [47], and NSCLC [48]. In contrast, we observed its decreased expression to be associated with worse prognosis in KIRC. Accordingly, APP may play dual roles in tumor progression and act as an antioncogene in KIRC.

To investigate the biological functions of the five-gene signature in KIRC, GO analysis and PCA were performed, demonstrating that the combined signature was able to distinguish different immune states, and the signature genes were mainly involved in immune-related processes; besides, immune response might serve as an underlying mechanism of the signature genes impacting KIRC progression. For treating patients with metastatic RCC, targeted therapies and immunotherapies such as high-dose IL-2 and immune checkpoint inhibitors (ICIs) have been introduced [49, 50]. Nevertheless, many patients fail to benefit from the therapy strategies [50, 51]. Given the association between the signature genes and immune states, these genes might be related to immune therapeutic response of KIRC. Scholars have combined preclinical RNA-Seq data with clinical gene expression profile to establish predictive signatures of prognosis and therapeutic response for gliomas [52, 53]. Inspired by their work, we would further use single-cell RNA-Seq data to explore the roles of the signature genes in immunotherapy.

## 5. Conclusions

In general, we performed a pan-cancer analysis of abnormally expressed and survival-related IRGs across 10 cancer types, indicating a strong correlation between IRGs and the prognosis of KIRC. We further established an IRGs signature that could independently predict survival for patients with KIRC, which confirmed the prognostic value of inflammasomes in KIRC. Moreover, the signature might influence the progression of KIRC. Further exploration on biological functions of the IRGs signature suggested that the signature genes are mostly involved in immune-related pathways and provided novel perspectives for therapy of KIRC. Thus, our study not only presented a systematic landscape of IRGs across human cancers but also developed a robust prognostic predictor for KIRC from the perspective of pan-cancer analysis.

## Figures and Tables

**Table 1:** Clinical characteristics of the KIRC patients.

Id	No. of KIRC samples	No. of normal renal tissue samples	KIRC samples				
			Death events	Mean age (years)	Gender (female/male)	Stage (I/II/III/IV)	Grade (1/2/3/4)
TCGA	526	72	170	60.42	183/343	261/57/123/82	13/226/205/74
GSE40435	101	101	NA	64.12	42/59	—	22/47/24/8
GSE53757	72	72	NA	NA	—	24/19/14/15	—
GSE73731	265	—	NA	NA	102/160	41/12/28/44	22/90/95/49

**Table 2:** Univariate and LASSO regression analyses of 8 IRGs in the entire TCGA set.

Symbol	Univariate regression		LASSO coefficient
	HR	*p* value	
PSTPIP1	1.4301	0.0004	—
IFI16	1.7617	0.0001	0.5409
NLRC5	1.2888	0.0165	—
IL1RL1	0.8195	0.0378	−0.0698
AIM2	1.6595	0.0001	0.1449
TXNIP	0.6896	0.0001	−0.3261
PYCARD	1.4824	0.0001	—
APP	0.5769	0.0001	−0.5283

**Table 3:** Univariate and multivariate Cox regression analyses of the IRGs signature in the entire TCGA set.

Variable		Univariate regression		Multivariate regression	
		HR	*p* value	HR	*p* value
Age (years)	≤65 vs. > 65	0.6065	0.0012	0.7141	0.1131
Gender	Female vs. male	1.0415	0.7990	—	—
T stage	T1-T2 vs. T3-T4	0.3067	0.0001	0.5932	0.0346
N stage	N0 vs. N1	0.3168	0.0007	0.8144	0.5783
M stage	M0 vs. M1	0.2210	0.0001	0.3555	0.0001
Grade	G1-G2 vs. G3-G4	0.3611	0.0001	0.5847	0.0412
Risk	High vs. low	2.8241	0.0001	2.0438	0.0041

## Data Availability

All raw data included in this study can be acquired from the two public repositories: The Cancer Genome Atlas (TCGA) database and Gene Expression Omnibus (GEO) database. And, the data used to support our findings are available from the corresponding author upon request.

## Supplementary Materials

Supplementary Materials The PPI network of the 40 IRGs is shown in Supplementary Figure 1. Supplementary Figure 2 presents the construction of LASSO Cox regression model, and each patient's risk score, survival status, and the five-gene expression.

## References

[1] Martinon F., Burns K., Tschopp J. (2002). The inflammasome. *Molecular Cell*.

[2] von Moltke J., Ayres J. S., Kofoed E. M., Chavarría-Smith J., Vance R. E. (2013). Recognition of bacteria by inflammasomes. *Annual Review of Immunology*.

[3] Vandanmagsar B., Youm Y.-H., Ravussin A. (2011). The NLRP3 inflammasome instigates obesity-induced inflammation and insulin resistance. *Nature Medicine*.

[4] Kahlenberg J. M., Kang I. (2019). *Clinicopathologic Significance of Inflammasome Activation in Autoimmune Diseases*.

[5] Ershaid N., Sharon Y., Doron H. (2019). NLRP3 inflammasome in fibroblasts links tissue damage with inflammation in breast cancer progression and metastasis. *Nature Communications*.

[6] Terme M., Ullrich E., Aymeric L. (2011). IL-18 induces PD-1-dependent immunosuppression in cancer. *Cancer Research*.

[7] Chow M. T., Sceneay J., Paget C. (2012). NLRP3 suppresses NK cell-mediated responses to carcinogen-induced tumors and metastases. *Cancer Research*.

[8] Sagulenko V., Thygesen S. J., Sester D. P. (2013). AIM2 and NLRP3 inflammasomes activate both apoptotic and pyroptotic death pathways via ASC. *Cell Death & Differentiation*.

[9] Sborgi L., Rühl S., Mulvihill E. (2016). GSDMD membrane pore formation constitutes the mechanism of pyroptotic cell death. *The EMBO Journal*.

[10] Shi J., Zhao Y., Wang K. (2015). Cleavage of GSDMD by inflammatory caspases determines pyroptotic cell death. *Nature*.

[11] Strowig T., Henao-Mejia J., Elinav E., Flavell R. (2012). Inflammasomes in health and disease. *Nature*.

[12] Drexler S. K., Bonsignore L., Masin M. (2012). Tissue-specific opposing functions of the inflammasome adaptor ASC in the regulation of epithelial skin carcinogenesis. *Proceedings of the National Academy of Sciences*.

[13] Deng Q., Geng Y., Zhao L. (2019). NLRP3 inflammasomes in macrophages drive colorectal cancer metastasis to the liver. *Cancer Letters*.

[14] Kolb R., Kluz P., Tan Z. W. (2019). Obesity-associated inflammation promotes angiogenesis and breast cancer via angiopoietin-like 4. *Oncogene*.

[15] Weinstein J. N., Lingua E. A., Collisson E. A. (2013). The Cancer Genome Atlas Pan-Cancer analysis project. *Nature Genetics*.

[16] Shuch B., Amin A., Armstrong A. J. (2015). Understanding pathologic variants of renal cell carcinoma: distilling therapeutic opportunities from biologic complexity. *European Urology*.

[17] Lin Y.-W., Lee L.-M., Lee W.-J. (2016). Melatonin inhibits MMP-9 transactivation and renal cell carcinoma metastasis by suppressing Akt-MAPKs pathway and NF-*κ*B DNA-binding activity. *Journal of Pineal Research*.

[18] MacLennan S., Imamura M., Lapitan M. C. (2012). Systematic review of perioperative and quality-of-life outcomes following surgical management of localised renal cancer. *European Urology*.

[19] Subramanian A., Tamayo P., Mootha V. K. (2005). Gene set enrichment analysis: a knowledge-based approach for interpreting genome-wide expression profiles. *Proceedings of the National Academy of Sciences*.

[20] Liberzon A., Birger C., Thorvaldsdóttir H., Ghandi M., Mesirov J. P., Tamayo P. (2015). The molecular signatures database hallmark gene set collection. *Cell Systems*.

[21] Wozniak M. B., Le Calvez-Kelm F., Abedi-Ardekani B. (2013). Integrative genome-wide gene expression profiling of clear cell renal cell carcinoma in Czech Republic and in the United States. *PLoS One*.

[22] von Roemeling C. A., Radisky D. C., Marlow L. A. (2014). Neuronal pentraxin 2 supports clear cell renal cell carcinoma by activating the AMPA-selective glutamate receptor-4. *Cancer Research*.

[23] Wei X., Choudhury Y., Lim W. K. (2017). Recognizing the continuous nature of expression heterogeneity and clinical outcomes in clear cell renal cell carcinoma. *Scientific Reports*.

[24] Ritchie M. E., Phipson B., Wu Di (2015). Limma powers differential expression analyses for RNA-sequencing and microarray studies. *Nucleic Acids Research*.

[25] McCarthy D. J., Chen Y., Smyth G. K. (2012). Differential expression analysis of multifactor RNA-Seq experiments with respect to biological variation. *Nucleic Acids Research*.

[26] Szklarczyk D., Gable A. L., Lyon D. (2019). STRING v11: protein-protein association networks with increased coverage, supporting functional discovery in genome-wide experimental datasets. *Nucleic Acids Research*.

[27] Shannon P., Markiel A., Ozier O. (2003). Cytoscape: a software environment for integrated models of biomolecular interaction networks. *Genome Research*.

[28] Raudvere U., Kolberg L., Kuzmin I. (2019). g:Profiler: a web server for functional enrichment analysis and conversions of gene lists (2019 update). *Nucleic Acids Research*.

[29] Friedman J., Hastie T., Tibshirani R. (2010). Regularization paths for generalized linear models via coordinate descent. *Journal of Statistical Software*.

[30] Gao M., Zhang P., Huang L. (2019). Is NLRP3 or NLRP6 inflammasome activation associated with inflammation-related lung tumorigenesis induced by benzo(a)pyrene and lipopolysaccharide?. *Ecotoxicology and Environmental Safety*.

[31] Flood B., Manils J., Nulty C. (2019). Caspase-11 regulates the tumour suppressor function of STAT1 in a murine model of colitis-associated carcinogenesis. *Oncogene*.

[32] Hua X., Chen J., Su Y. (2020). Identification of an immune-related risk signature for predicting prognosis in clear cell renal cell carcinoma. *Aging*.

[33] Zeng J.-H., Lu W., Liang L. (2019). Prognosis of clear cell renal cell carcinoma (ccRCC) based on a six-lncRNA-based risk score: an investigation based on RNA-sequencing data. *Journal of Translational Medicine*.

[34] Chen L., Luo Y., Wang G. (2019). Prognostic value of a gene signature in clear cell renal cell carcinoma. *Journal of Cellular Physiology*.

[35] Hornung V., Ablasser A., Charrel-Dennis M. (2009). AIM2 recognizes cytosolic dsDNA and forms a caspase-1-activating inflammasome with ASC. *Nature*.

[36] Unterholzner L., Keating S. E., Baran M. (2010). IFI16 is an innate immune sensor for intracellular DNA. *Nature Immunology*.

[37] DeYoung K. L., Ray M. E., Su Y. A. (1997). Cloning a novel member of the human interferon-inducible gene family associated with control of tumorigenicity in a model of human melanoma. *Oncogene*.

[38] Man S. M., Zhu Q., Zhu L. (2015). Critical role for the DNA sensor AIM2 in stem cell proliferation and cancer. *Cell*.

[39] Fujiuchi N., Aglipay J. A., Ohtsuka T. (2004). Requirement of IFI16 for the maximal activation of p53 induced by ionizing radiation. *Journal of Biological Chemistry*.

[40] Lin W., Zhao Z., Ni Z. (2017). IFI16 restoration in hepatocellular carcinoma induces tumour inhibition via activation of p53 signals and inflammasome. *Cell Proliferation*.

[41] Zhang M., Jin C., Yang Y. (2019). AIM2 promotes non-small-cell lung cancer cell growth through inflammasome-dependent pathway. *Journal of Cellular Physiology*.

[42] Kondo Y., Nagai K., Nakahata S. (2012). Overexpression of the DNA sensor proteins, absent in melanoma 2 and interferon-inducible 16, contributes to tumorigenesis of oral squamous cell carcinoma with p53 inactivation. *Cancer Science*.

[43] Viana C. T. R., Orellano L. A. A., Pereira L. X. (2018). Cytokine production is differentially modulated in malignant and non-malignant tissues in ST2-receptor deficient mice. *Inflammation*.

[44] Nagaraj K., Lapkina-Gendler L., Sarfstein R. (2018). Identification of thioredoxin-interacting protein (TXNIP) as a downstream target for IGF1 action. *Proceedings of the National Academy of Sciences*.

[45] Gao Y., Qi J.-C., Li X. (2020). Decreased expression of TXNIP predicts poor prognosis in patients with clear cell renal cell carcinoma. *Oncology Letters*.

[46] Tsang J. Y. S., Lee M. A., Chan T.-H. (2018). Proteolytic cleavage of amyloid precursor protein by ADAM10 mediates proliferation and migration in breast cancer. *EBioMedicine*.

[47] Hansel D. E., Rahman A., Wehner S. (2003). Increased expression and processing of the Alzheimer amyloid precursor protein in pancreatic cancer may influence cellular proliferation. *Cancer Research*.

[48] Sobol A., Galluzzo P., Weber M. J., Alani S., Bocchetta M. (2015). Depletion of amyloid precursor protein (APP) causes G0 arrest in non-small cell lung cancer (NSCLC) cells. *Journal of Cellular Physiology*.

[49] Garje R., An J., Greco A. (2020). The future of immunotherapy-based combination therapy in metastatic renal cell carcinoma. *Cancers*.

[50] Flippot R., Escudier B., Albiges L. (2018). Immune checkpoint inhibitors: toward new paradigms in renal cell carcinoma. *Drugs*.

[51] Rini B. I., McDermott D. F., Hammers H. (2016). Society for immunotherapy of cancer consensus statement on immunotherapy for the treatment of renal cell carcinoma. *Journal for Immunotherapy of Cancer*.

[52] Zhang J., Guan M., Wang Q. (2019). Single-cell transcriptome-based multilayer network biomarker for predicting prognosis and therapeutic response of gliomas. *Briefings in Bioinformatics*.

[53] Sun X., Liu X., Xia M. (2019). Multicellular gene network analysis identifies a macrophage-related gene signature predictive of therapeutic response and prognosis of gliomas. *Journal of Translational Medicine*.

