# Supplementary material for: Prognostic Inflammasome-Related Signature Construction in Kidney Renal Clear Cell Carcinoma Based on a Pan-Cancer Landscape
Source: Evid Based Complement Alternat Med. 2020 Apr 3;2020:3259795. doi: 10.1155/2020/3259795 (PMC7157792; doi:10.1155/2020/3259795)
Supplement: Supplementary Materials — The PPI network of the 40 IRGs is shown in Supplementary Figure 1. Supplementary Figure 2 presents the construction of LASSO Cox regression model, and each patient's risk score, survival status, and the five-gene expression. [file 3259795.f1.docx]

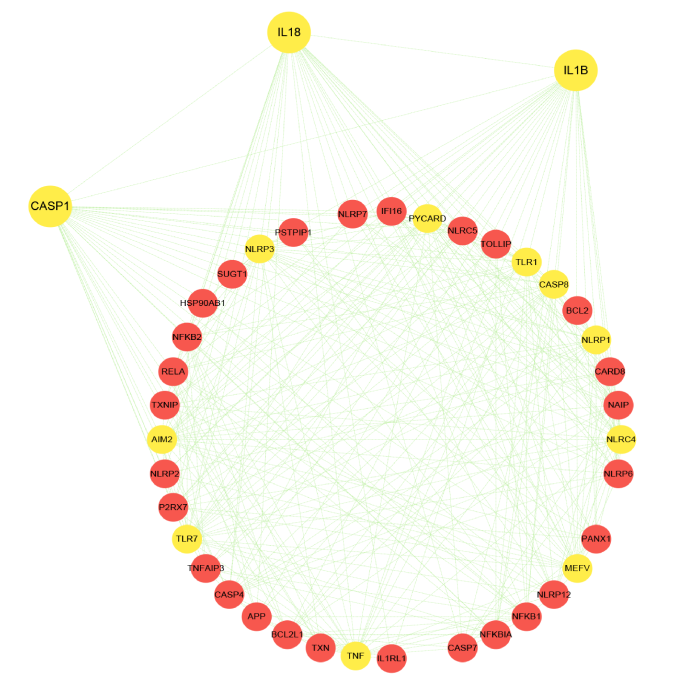


**Supplementary Figure 1:** PPI network of IRGs, and yellow represents the degree≥20.


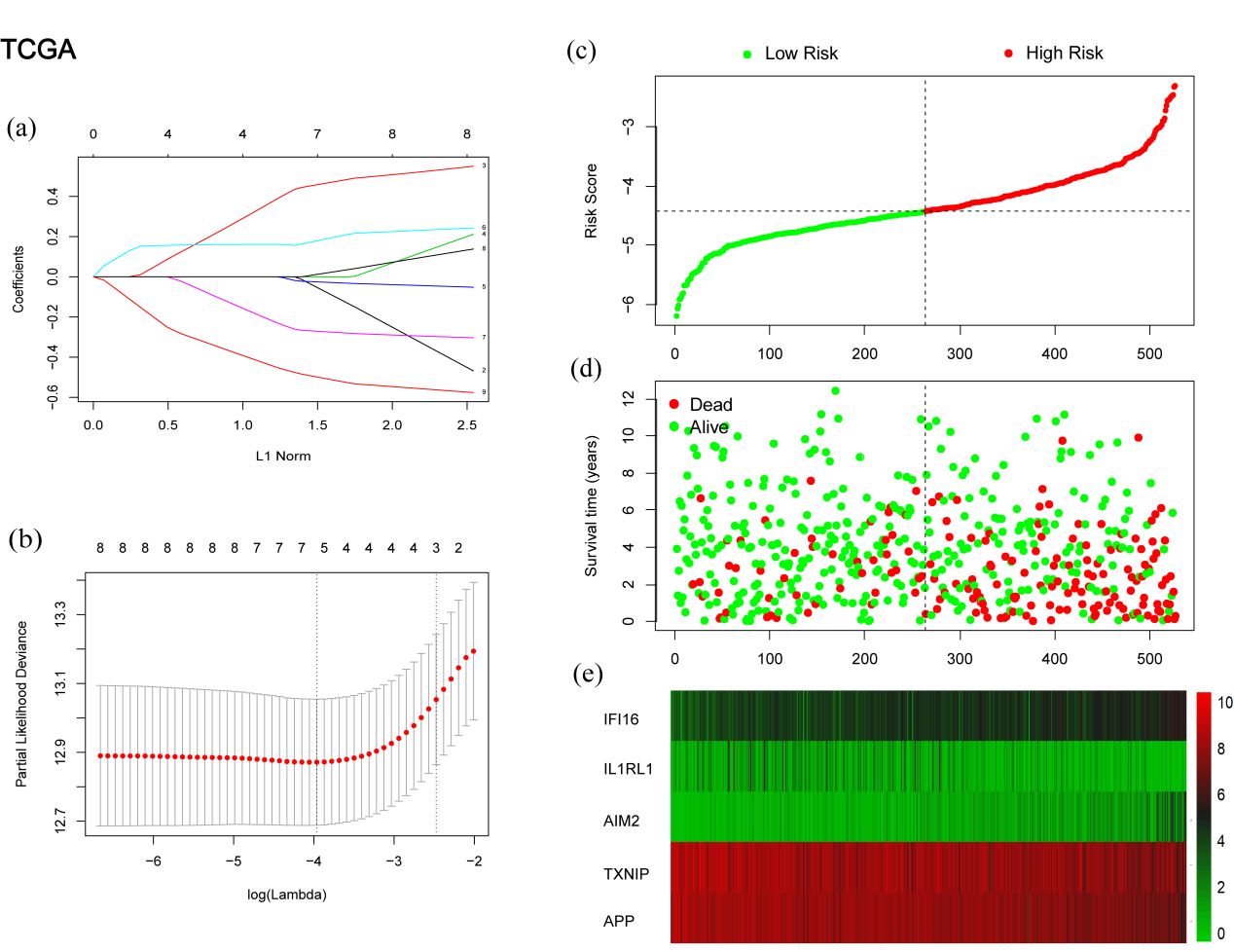


**Supplementary Figure 2:** (a)-(b) Construction of LASSO Cox regression model based on prognostic dysregulated IRGs in the entire TCGA set. (c)-(d) Relationship between survival time (years) and risk score rank in the entire TCGA set. (e) The prognostic five-gene expression of the high and low-risk groups in the entire TCGA set.
